# Characteristics and associated factors of violence in male patients with schizophrenia in China

**DOI:** 10.3389/fpsyt.2023.1106950

**Published:** 2023-03-10

**Authors:** Weilong Guo, Yu Gu, Jiansong Zhou, Xiaoping Wang, Qiaoling Sun

**Affiliations:** ^1^Department of Psychiatry, The Second Xiangya Hospital, Central South University, Changsha, Hunan, China; ^2^Hunan Key Laboratory of Psychiatry and Mental Health, China National Clinical Research Center on Mental Disorders (Xiangya), China National Technology Institute on Mental Disorders, Hunan Technology Institute of Psychiatry, Mental Health Institute of Central South University, Changsha, Hunan, China

**Keywords:** schizophrenia, associated factor, male, violence, China

## Abstract

**Objective:**

To investigate the characteristics and associated factors of violence in male patients with schizophrenia in China.

**Methods:**

A total of 507 male patients with schizophrenia were recruited, including 386 non-violent and 121 violent patients. The socio-demographic information and medical history of the patients were collected. Psychopathological characteristics, personality traits psychopathology, and factors related to risk management were assessed using the Brief Psychiatric Rating Scale (BPRS), the History of Violence, Clinical, Risk Assessment Scale (HCR-20), and the Psychopathy Checklist-Revised (PCL-R), as appropriate. Differences in these factors were compared between the violent and non-violent patients, and logistic regression analysis was performed to explore the risk factors for violence in male patients with schizophrenia.

**Results:**

The results showed that the violent group had a lower level of education, longer duration of illness, as well as a higher rate of hospitalization, history of suicidal attempts, and history of alcohol compared with the non-violent group. The violent group scored higher in items of symptoms in BPRS, personality traits and psychopathy in PCL-R, and risk management in HCR-20. The regression analysis showed that previous suicidal behavior (OR = 2.07,95% CI [1.06-4.05], *P* = 0.033), antisocial tendency in PCL-R (OR = 1.21, 95% CI [1.01-1.45], *P* = 0.038), H2: young age at violent incident (OR = 6.39, 95% CI [4.16-9.84], *P* < 0.001), C4: impulsivity (OR = 1.76, 95% CI [1.20-2.59], *P* = 0.004), and H3: relationship instability (OR = 1.60, 95% CI [1.08-2.37], *P* = 0.019) in HCR-20 were risk factors of violence among male patients with schizophrenia.

**Conclusion:**

The present study found significant differences in socio-demographic information, history of treatment, and psychopathy characteristics between male patients with schizophrenia who had engaged in violent behaviors and their non-violent counterparts in China. Our findings suggested the necessity of individualized treatment for male patients with schizophrenia who had engaged in violent behaviors as well as the use of both HCR-20 and PCL-R for their assessment.

## Introduction

People with schizophrenia have increased risks for a range of adverse outcomes, including violent outcomes, compared with the general population (1, 2). A 38-year follow-up study on 24,297 patients with schizophrenia and related disorders found that 2.7% of the female patients and 10.7% of the male patients were convicted of violent crimes within 5 years after their first diagnosis (2). An 18-month follow-up study on 1,435 patients with schizophrenia who participated in a clinical trial found that 77 (5.4%) of the participants had engaged in violent behaviors (3). Although most patients with schizophrenia never display any violent behaviors in their lifetime, this population may be at a higher risk of violence compared to the general population. A meta-analysis showed that, compared with the general population, male patients with schizophrenia had a four-fold increased risk of violent crime, and female patients were at an eight-fold increased risk (1). Lately, through a birth cohort study in Switzerland, Fazel et al. reported that the risk of violence in patients with schizophrenia was 10.7 times that of the general population and 4.2 times that of their unaffected siblings (4).

Related factors for violence have always been the focus and hotspot in the field of forensic psychiatry. It is believed that social and demographic factors such as age, level of education, and economic status are associated with the risk of violence among patients with schizophrenia. Patients at a younger age and with a lower socioeconomic status (e.g., those with a lower level of education or a lower income) are at a higher risk of engaging in violent behaviors (2, 5, 6). Violence history is also regarded as a strong predictor of the risk of violence in patients with schizophrenia (2, 7). Furthermore, family environmental factors, such as child abuse and parents with substance abuse/history of crimes, have also been found to be associated with violent behaviors (8, 9). Clinically significant personality traits or psychopathy also seemed to be predictive of violence as well (10, 11). Psychoactive substance abuse is also an important associated factor for violence (12–14).

The associations between psychopathological and treatment factors and violence have also been a focus in studies of mental disorders. Numerous studies have found that positive symptoms are associated with an increased risk of violence in people with schizophrenia (15, 16). Among the positive symptoms, delusions are found to be the most closely associated with violent behaviors (16, 17), followed by hallucinations, especially commanding hallucination (18). The relation between negative symptoms and violence is still unclear. Although some literature reviews did not support the association between negative symptoms and violent behaviors (6), Swanson et al. found that a medium PANSS score of negative symptoms was associated with a lower risk of severe violence (19). Treatment factors, as modifiable factors in clinical settings, are also regarded as important factors related to violence in patients with schizophrenia. Factors such as duration of untreated illness (20, 21), treatment with antipsychotic medications (22, 23) and adherence (24) may affect the risk of violence among patients.

To date, there has been numerous valuable findings on the factors associated with violent behaviors in patients with schizophrenia. However, most of the studies were performed in Western countries; there are relatively fewer studies in China (25), and most of them had a small sample size or did not use standardized assessment tools. Due to the differences in the legal system, crime rate, and cultural background between China and Western countries, there might be disparities in the composition of violence-related factors. Therefore, it is necessary to systematically explore related characteristics and correlates among violent patient with schizophrenia in China.

In the present study, we aim to explore the characteristics and correlates of violence in patients with schizophrenia in China in the hope of providing a basis for the identification and intervention for violence is this patient group.

## Materials and methods

### Participants

This study adopted a case-control design. Considering that the incidence of violence varies significantly between genders, we only recruited male patients with schizophrenia. All the participants were inpatients and recruited from the psychiatric ward of the Second Xiangya Hospital of Central South University in Changsha, Hunan, from May 2013 to December 2021. Patients meeting the following criteria could be included: (1) male patients aged ≥ 18 years; (2) meeting the criteria for schizophrenia in The International Statistical Classification of Diseases and Related Health Problems 10th Revision (ICD-10); (3) able to understand the content of this study, voluntarily participating in the study and cooperative with assessments; and (4) the guardian of the patient signed the informed consent form. Patients meeting any of the following criteria were excluded: (1) unable to cooperate with or complete the assessments; (2) with comorbid mental disorders; and (3) with severe physical diseases or in a critical condition. Patients were divided into the violent group and the non-violent group based on their violence history, which was defined in this study as the intentional or unprepared acts against others, which caused physical harm to the victims for a total of at least 3 times. The study was reviewed and approved by the Ethics Committees of the Second Xiangya Hospital.

### Assessment tools

Socio-demographic and treatment factors were collected through a specific form, including age, level of education, duration of illness, treatment history, etc. The tools for assessment are as follows:

The Brief Psychiatric Rating Scale (BPRS) (26): BPRS is a 18-item scale used to evaluate psychopathological factors, with all items rated on a 7-point Likert scale. The Chinese version of BPRS has good reliability and validity (27, 28).

The Historical, Clinical, Risk management-20 items (HCR-20) Scale (29): HCR-20 is currently a more commonly used assessment tool for violence in the world and is applicable to patients with mental disorders or personality disorders. HCR-20 consists of 20 items, including three factors, i.e., historical factors, clinical factors, and management factors. Each item was rated on a 3-point Likert scale (scored 0-2). The Chinese version of HCR-20 has good short- and medium-term predictive validity (30).

Psychopathic Checklist-Revised (PCL-R) (31): PCL-R is mainly used to assess psychopathy. This 20-item scale includes four factors, i.e., interpersonal, affective, lifestyle, and antisocial factors, with each item rated on a 3-point Likert scale (scored 0-2) and a total score ranged 0-40. The PCL-R has been translated into Chinese and revised, and the revised version is found to have good internal consistency (32).

### Statistical analysis

Normally distributed continuous data were presented as mean ± standard deviation and evaluated by independent-sample t test. Duration of illness, which was not normally distributed, was analyzed using the Mann-Whitney U test. Categorical variables were presented as numbers and percentages and analyzed using Chi-squared test. If the theoretical frequency in any cell is lower than 5, Fisher’s exact test would be used. In order to identify factors correlated with violence behaviors, binomial logistic regression analysis was conducted. Variables that were statistically significant in the univariate analysis were entered into the ordinal logistic regression (with duplicated contents removed). All the analyses were performed using SPSS (Version 23; IBM, Inc., Chicago, Illinois), and all the statistical tests were two-sided and a *P*-value lower than 0.05 indicated statistical significance.

## Results

### Social-demographic and treatment characteristics

A total of 507 male patients with schizophrenia were recruited, including 386 non-violent patients and 121 violent patients. The social-demographic and treatment variables for different groups are shown in Tables 1, 2. The violent patients had a lower level of education (χ^2^ = 9.039, *P* = 0.029), longer duration of illness (*P* = 0.009), higher likelihood of having a history of alcohol use (χ^2^ = 4.612, *P* = 0.032) and higher likelihood of having a history of suicidal attempts (χ^2^ = 8.992, *P* = 0.003). Furthermore, the two groups also differed significantly in treatment history (χ^2^ = 7.283, *P* = 0.026), with a higher rate of hospitalization in the violent group (80.2 vs. 70.7%).

**TABLE 1:** Social and demographic characteristics of violent and non-violent patients with schizophrenia.

	Total (*n* = 507)	Non-violent group (*n* = 386)	Violent group (*n* = 121)	*P*
Age (year)	31.9 (11.38)	31.8 (11.56)	32.2 (10.82)	0.684^a^
Marital status				0.108^b^
Single	362 (71.4%)	277 (71.8%)	85 (70.2%)	
Married/partnered	104 (20.5)	83 (21.5%)	21 (17.4%)	
Divorced	41 (8.1%)	26 (6.7%)	15 (12.4%)	
Ethnicity				1.00^c^
Han	492 (97.0%)	374 (96.9%)	118 (97.5%)	
Others	15 (3.0%)	12 (3.1%)	3 (2.5%)	
Level of education				**0**.**029^b^**
Primary school or below	21 (4.1%)	15 (3.9%)	6 (5.0%)	
Secondary school	153 (30.2%)	111 (28.8%)	42 (34.7%)	
High school/specialized secondary school	186 (36.7%)	135 (35.0%)	51 (42.1%)	
College and above	147 (29.0%)	125 (32.4%)	22 (18.2%)	
Residence				0.051^b^
Urban	327 (64.5%)	240 (62.2%)	87 (71.9%)	
Rural	180 (35.5%)	146 (37.8%)	34 (28.1%)	
Employment				0.922^b^
Unemployed	262 (51.7%)	199 (51.6%)	63 (52.1%)	
Employed	245 (48.3%)	187 (48.4%)	58 (47.9%)	
Living status				0.638^b^
Alone	58 (11.4%)	45 (11.7%)	13 (10.7%)	
Core family	386 (76.1%)	296 (76.7%)	90 (74.4%)	
Others	63 (12.4%)	45 (11.7%)	18 (14.9%)	

^a^*t*-test, ^b^Chi-square test, ^c^Fisher’s exact test, *P*-values < 0.05 were marked in bold.

**TABLE 2:** Historical and environmental factors and treatment factors for violent and non-violent patients with schizophrenia.

	Total (*n* = 507)	Non-violent group (*n* = 386)	Violent group (*n* = 121)	*P*
Age of onset	23.44 (7.46)	23.66 (7.60)	22.76 (6.98)	0.248
Duration of illness (month)	97.49 (100.40)	92.37 (98.23)	113.76 (105.79)	**0**.**009^d^**
Treatment history				**0**.**026^b^**
Untreated	40 (7.9%)	37 (9.6%)	3 (2.5%)	
Outpatient	97 (19.1%)	76 (19.7%)	21 (17.4%)	
Inpatient	370 (73.0%)	273 (70.7%)	97 (80.2%)	
Number of previous hospitalizations				0.051^b^
0	137 (27.0%)	113 (29.3%)	24 (19.8%)	
1	107 (21.1%)	86 (22.3%)	21 (17.4%)	
2-3	133 (26.2%)	95 (24.6%)	38 (31.4%)	
> 3	130 (25.6%)	92 (23.8%)	38 (31.4%)	
History of craniocerebral injury				0.160^b^
No	485 (95.7%)	372 (96.4%)	113 (93.4%)	
Yes	22 (4.3%)	14 (3.6%)	8 (6.6%)	
History of craniocerebral diseases				**0**.**042^c^**
No	494 (97.4%)	380 (98.4%)	115 (95%)	
Yes	13 (2.6%)	6 (1.6%)	6 (5%)	
Chronic physical diseases				0.584^c^
Absent	488 (96.3%)	370 (95.9%)	118 (97.5%)	
Present	19 (3.7%)	16 (4.1%)	3 (2.5%)	
History of alcohol use				**0**.**032^b^**
No	350 (69%)	276 (71.5%)	74 (61.2%)	
Yes	157 (31%)	110 (28.5%)	47 (38.8%)	
History of other substance abuse				0.094^c^
No	488 (96.3%)	375 (97.2%)	113 (93.4%)	
Yes	19 (3.7%)	11 (2.8%)	8 (6.6%)	
History of suicidal behavior				
No	429 (84.6%)	337 (87.3%)	92 (76.0%)	
Yes	78 (15.4%)	49 (12.7%)	29 (24.0%)	
Family history				0.207^b^
No	326 (64.3%)	254 (65.8%)	72 (59.5%)	
Yes	181 (35.7%)	132 (34.2%)	49 (40.5%)	

^a^*t*-test, ^b^Chi-square test, ^c^Fisher’s exact test, ^d^Mann-Whitney U test, *P*-values < 0.05 were marked in bold.

### Comparison of BPRS, HCR-20 and PCL-R scores

The violent patients had a higher BPRS total score (*t* = −2.407, *P* = 0.017) and a higher score of hostility-suspicion (*t* = −3.741, *P* < 0.001), as compared with the non-violent patients (Table 3). Compared with the non-violent group, the violent group had a higher score in conceptual disorganization (3.67 vs. 3.23, *P* = 0.007), mannerism and posturing (1.93 vs. 1.65, *P* = 0.015), hostility (3.20 vs. 2.53, *P* < 0.001), suspiciousness (4.38 vs. 3.65, *P* < 0.001), and disorientation (1.46 vs. 1.29, *P* = 0.001), and a lower score in motor retardation (1.67 vs. 2.02, *P* = 0.019). No significant differences were found between the two groups regarding the remaining items (Table 3).

**TABLE 3:** Total score, scores of all factor and each item of BPRS in violent and non-violent patients with schizophrenia.

	Total (*n* = 507)	Non-violent group (*n* = 386)	Violent group (*n* = 121)	*P*
Total BPRS score	41.91 (10.94)	41.33 (11.39)	43.77 (9.15)	**0.017**
**Factor BPRS score**				
Anxiety-depression	7.33 (3.18)	7.30 (3.23)	7.41 (3.02)	0.759
Withdrawal	9.61 (3.71)	9.58 (3.87)	9.72 (3.17)	0.680
Thinking disorder	11.10 (3.72)	10.96 (3.83)	11.54 (3.31)	0.112
Activity	5.28 (2.24)	5.21 (2.23)	5.49 (2.30)	0.239
Hostility-suspicion	8.60 (3.50)	8.28 (3.53)	9.62 (3.20)	**< 0.001**
**Items**				
Somatic concern	1.93 (1.31)	1.96 (1.31)	1.85 (1.30)	0.292
Anxiety	2.25 (1.36)	2.22 (1.37)	2.36 (1.31)	0.261
Emotional withdrawal	3.21 (1.42)	3.17 (1.44)	3.34 (1.36)	0.278
Conceptual disorganization	3.34 (1.75)	3.23 (1.74)	3.67 (1.78)	**0.007**
Guilt feelings	1.29 (0.80)	1.27 (0.77)	1.33 (0.90)	0.822
Tension	2.12 (1.28)	2.11 (1.30)	2.17 (1.23)	0.570
Mannerism and posturing	1.72 (1.05)	1.65 (1.01)	1.93 (1.14)	**0.015**
Grandiosity	1.42 (1.03)	1.44 (1.06)	1.36 (0.95)	0.529
Depressive moods	1.86 (1.21)	1.85 (1.18)	1.87 (1.30)	0.857
Hostility	2.69 (1.55)	2.53 (1.53)	3.20 (1.51)	**<0.001**
Suspiciousness	3.82 (1.76)	3.65 (1.78)	4.38 (1.61)	**<0.001**
Hallucinations	3.01 (1.92)	3.00 (1.88)	3.06 (2.03)	0.878
Motor retardation	1.94 (1.25)	2.02 (1.31)	1.67 (0.99)	**0.019**
Uncooperativeness	2.09 (1.40)	2.10 (1.41)	2.04 (1.38)	0.682
Unusual thought content	3.33 (1.62)	3.30 (1.67)	3.45 (1.46)	0.510
Blunted affect	3.13 (1.53)	3.10 (1.57)	3.25 (1.41)	0.312
Excitement	1.44 (0.97)	1.45 (1.00)	1.40 (0.86)	0.934
Disorientation	1.33 (0.80)	1.29 (0.80)	1.46 (0.81)	**0.001**

*P*-values < 0.05 were marked in bold.

The violent patients had higher scores in all subscales of HCR-20 (Table 4). Compared with the non-violent group, the violent group scored significantly higher with regard to the following items in the historical subscale: H2 – young age at first violent incident, H3 - relationship instability, H4 - employment problems, H8 - early maladjustment, H9 - personality disorder and H10 - prior release or detention failure. The violent group also scored significantly higher in the following items in the clinical subscale: C2 - negative attitudes, C4 - impulsivity, and C5 - unresponsive to treatment. For the risk management subscale, the violent group scored significantly higher in all the five items (Table 4).

**TABLE 4:** Total score, scores of all factor and each items of HCR-20 in violent and non-violent patients with schizophrenia.

	Total (*n* = 507)	Non-violent group (*n* = 386)	Violent group (*n* = 121)	*P*
HCR-20	14.35 ± 5.71	12.83 ± 5.03	19.22 ± 4.97	**<0**.**001**
H score	6.05 ± 2.75	5.17 ± 2.25	8.84 ± 2.31	**<0**.**001**
C score	5.38 ± 2.06	5.05 ± 2.03	6.44 ± 1.78	**<0**.**001**
R score	2.92 ± 2.22	2.61 ± 2.12	3.93 ± 2.25	**<0**.**001**
H1: Previous violence	0.75 ± 0.82	0.35 ± 0.48	2.00 ± 0.00	−
H2: Young age at first violent incident	0.61 ± 0.721	0.39 ± 0.62	1.31 ± 0.55	**<0**.**001**
H3: Relationship instability	0.90 ± 0.77	0.79 ± 0.75	1.26 ± 0.71	**<0**.**001**
H4: Employment problems	1.04 ± 0.88	0.98 ± 0.88	1.21 ± 0.84	**0**.**012**
H5: Substance use problems	0.10 ± 0.41	0.12 ± 0.45	0.05 ± 0.25	0.236
H6: Major mental illness	2.00 ± 0.00	2.00 ± 0.00	2.00 ± 0.00	1.000
H7: Psychopathy	0.27 ± 0.60	0.25 ± 0.61	0.31 ± 0.58	0.077
H8: Early maladjustment	0.28 ± 0.62	0.22 ± 0.55	0.48 ± 0.75	**<0**.**001**
H9: Personality disorder	0.06 ± 0.27	0.04 ± 0.23	0.15 ± 0.38	**<0**.**001**
H10: Prior release or detention failure	0.04 ± 0.25	0.02 ± 0.18	0.08 ± 0.36	**0**.**020**
C1: Lack of insight	1.60 ± 0.61	1.59 ± 0.63	1.63 ± 0.55	0.910
C2: Negative attitudes	0.65 ± 0.79	0.57 ± 0.76	0.92 ± 0.82	**<0**.**001**
C3: Active symptoms of major mental illness	1.68 ± 0.58	1.65 ± 0.61	1.76 ± 0.50	0.100
C4: Impulsivity	0.75 ± 0.78	0.59 ± 0.72	1.26 ± 0.76	**<0**.**001**
C5: Unresponsive to treatment	0.70 ± 0.59	0.64 ± 0.60	0.88 ± 0.53	**<0**.**001**
R1: Plans lack feasibility	0.74 ± 0.74	0.66 ± 0.74	0.98 ± 0.70	**<0**.**001**
R2: Exposure to destabilizers	0.58 ± 0.63	0.52 ± 0.61	0.79 ± 0.62	**<0**.**001**
R3: Lack of personal support	0.35 ± 0.61	0.30 ± 0.59	0.51 ± 0.65	**<0**.**001**
R4: Non-compliance with remediation attempts	0.81 ± 0.73	0.75 ± 0.74	0.99 ± 0.68	**0**.**001**
R5: Stress	0.45 ± 0.56	0.38 ± 0.54	0.67 ± 0.57	**<0**.**001**

H-Historical; C-Clinical; R-Risk Management. *P*-values < 0.05 were marked in bold.

The total score and scores of all factors in PCL-R are presented in Table 5. The result showed that the total score of PCL-R and scores of all factors were significantly higher in the violent group, as compared to the non-violent group (*p* < 0.05).

**TABLE 5:** The total score and scores of all factors of PCL-R in violent and non-violent patients with schizophrenia.

	Total (*n* = 507)	Non-violent group (*n* = 386)	Violent group (*n* = 121)	Statistics	*P*
Total score	8.31 (6.39)	6.93 (5.78)	12.22 (6.68)	−7.840	**<0.001**
Interpersonal score	1.02 (1.48)	0.89 (1.39)	1.45 (1.69)	−3.287	**0.001**
Affective score	2.59 (2.50)	2.15 (2.26)	3.98 (2.71)	−6.741	**<0.001**
Lifestyle score	3.35 (2.70)	2.93 (2.57)	4.69 (2.67)	−6.500	**<0.001**
Antisocial score	1.65 (1.65)	1.30 (1.46)	2.75 (1.74)	−9.092	**<0.001**

*P*-values < 0.05 were marked in bold.

### Risk factors of violence

After removal of the duplicated factors, the factors with significant inter-group difference were included in the logistic regression analysis as independent variables. The included variables and types of these variables are presented in Supplementary Table 1. After 19 times of stepwise analysis with the use of the backward LR method, it was found that the history of suicidal attempts (OR = 2.07,95% CI [1.06-4.05], *P* = 0.033), antisocial tendency in PCL-R (OR = 1.21, 95% CI [1.01-1.45], *P* = 0.038), H2: young age at violent incident (OR = 6.39, 95% CI [4.16-9.84], *P* < 0.001), C4: impulsivity (OR = 1.76, 95% CI [1.20-2.59], *P* = 0.004), and H3: relationship instability (OR = 1.60, 95% CI [1.08-2.37], *P* = 0.019) were associated with an increased risk of violence (see Table 6).

**TABLE 6:** Result of the logistic regression analysis.

Factor	OR	95% CI	*P*
History of suicidal behavior	2.07	1.06-4.05	**0**.**033**
PCL-R antisocial factor	1.21	1.01-1.45	**0**.**038**
Young age at violent incident (H2)	6.39	4.16-9.84	**<0**.**001**
Impulsivity (C4)	1.76	1.20-2.59	**0**.**004**
Relationship instability (H3)	1.60	1.08-2.37	**0**.**019**

*P*-values < 0.05 were marked in bold.

## Discussion

Using male patients with schizophrenia as subjects, the present study investigated the differences in socio-demographic characteristics, historical and environmental factors, and treatment factors between violent and non-violent patient with schizophrenia; we also further examined the potential risk factors of violence using logistic regression analysis. Our findings showed that, compared with the non-violent group, the violent group had a lower level of education, longer duration of illness, as well as a higher rate of hospitalization, history of suicidal attempts, and history of alcohol. The violent group scored higher in items of symptoms in BPRS, personality traits and psychopathy in PCL-R, and risk management in HCR-20. The regression analysis suggested that history of suicidal attempts, young age at violent incident (H2), impulsivity (C4) and relationship instability (H3) in HCR-20, and the antisocial factor in PCL-R were risk factors of violent behaviors. These findings may provide us with important reference for the treatment and management of male patients with schizophrenia and the necessity of using both HCR-20 and PCL-R for the assessment of these patients.

The present study found that a lower level of education has been deemed as associated with an increased risk of violence. Similar to previous studies (33), the violent patients in the present study had a lower level of education. The education status could indirectly reflect the conditions of patients with schizophrenia: patients with more severe condition or an early onset were less likely to continue their education, and a lower level of education might in turn reflect poorer social functions.

Our findings showed that violent patients have longer duration of illness and a higher rate of hospitalization. This could be explained by the long course of disease *per se* increasing the likelihood of hospitalization. The occurrence of violent behaviors may also be one of the reasons for hospitalization, as the parents or other guardians of the patients might think it could help them avoid harm from the patients. Multiple times of hospitalization are also considered as indicators of severe disease and non-compliance with treatment. This may imply the role of sufficient treatment and improved medication compliance in reducing the risk of violence in male patients with schizophrenia.

The present study showed that the violent patients were more likely to have a history of suicidal attempts, and the regression analysis showed that the history of suicidal attempts was a risk factor of violence. Although suicidal attempts and violence are regarded as two different outcomes, some studies suggested that the two phenomena often co-occurred in the general population and patients with mental disorders (34, 35). The association between suicidal attempts and violence might be mediated by shared risk factors (36), such as age, alcohol consumption, and impulsivity. Moreover, the violent patients were more likely to have a history of alcohol use, while the subsequent analysis did not reveal a significant association between the history of alcohol use and violence, which is not in line with other findings (2, 4). A possible explanation is that simple alcohol consumption (without meeting the criteria for alcohol abuse or alcohol use disorder) may not affect the occurrence of violence greatly. In the present study, few subjects had a history of other substance abuse, and no statistical difference was found between groups. As the incidence of substance abuse is relatively low and people are likely to hide their history of substance use in China (37), this study might have underestimated the impact of substance abuse on violence. It is widely believed that violence is related to substance abuse (1, 38), and some studies even suggested that substance abuse was a major risk factor of violence among patients with schizophrenia (1). Although the incidence of psychoactive substance abuse in China is significantly lower than that in some Western countries, the detection and management of substance abuse is still essential for patients in China.

The present study found that violent patients scored higher in items related to hostility, suspiciousness, and conceptual disorganization in BPRS, which was consistent with previous studies. Among untreated patients with schizophrenia in prison, persecutory delusion was found to be significantly associated with the risk of violence (17). A clinical study on the effect of antipsychotic agents suggested that hallucinations with delusional interpretation and delusional thoughts related to suspicions and sense of being persecuted were associated with an increased risk of violence among patients with schizophrenia (19). In a cross-sectional study on inpatients, Steinert et al. found that thought disorders are strongly associated with increased violent behaviors (39). However, the regression analyses did not reveal a significant association between psychotic symptoms and violence. The disparity might be explained as follows. First, psychotic symptoms do not directly increase the risk of violence, but they may be mediated by other factors such as anger, which, if caused by delusional beliefs, may be a key factor for the association between psychotic disorders and violence (40). This may explain why many patients with schizophrenia who had severe psychotic symptoms do not become violent, which has been observed in clinical practice. Second, fluctuations in symptoms, rather than the symptoms themselves, may contribute to the increased risk of violence (41).

In the present study found that violent patients scored higher in items of lack of personal support (R3), plans lack feasibility (R1), non-compliance with remediation attempts (R4), and stress (R5) in HCR-20, which was consistent with previous findings. Nicholls et al. found that, in both the hospital environment and the community environment, the risk management factor in HCR-20 was predictive of violent behaviors for both male and female patients (42). In addition, higher scores in personality disorder (H9) in HCR-20 and higher total score and the scores of all factors in PCL-R were found in the violent group, who were not comorbid with personality disorders. This is possible as the item H9 was used to measure, rather than diagnose, personality trait. In view of the previous studies finding that PCL-R could predict violence, and this predictive effect remained significant after controlling for the effects of substance abuse (11), the personality traits may also be taken into account in the treatment and management of patients. The regression analysis suggested that the antisocial factor in PCL-R and young age at violent incident (H2), impulsivity (C4) and relationship instability (H3) in HCR-20 were risk factors of violence in schizophrenia, suggesting the value of these assessment tools for patients with male patients with schizophrenia.

In the present study, there were significant differences in socio-demographics, history of treatment, and psychopathy characteristics between the violent patients and those without a history of violent behaviors. This may imply that schizophrenia with aggressive or violent behaviors may be a subtype of this disorder. Patients with preexisting conduct problems may have antisocial attitudes and thinking patterns and lack appropriate social skills from an early age, and they may have less neurological damage than patients with schizophrenia (43, 44). The pattern of aggression or violence in these patients may differ from that in those whose aggression or violence occurred after the onset of schizophrenia (45). All subtypes of patients need to take antipsychotics, but for patients with antisocial behaviors, attitudes, and thinking patterns, the education about their illness and the need for medication is a major challenge for healthcare workers. Comprehensive assessment and individualized rehabilitation may be helpful, to some extent, to address this issue. Meanwhile, more studies are also needed to verify this hypothesis in the future.

Despite the strengths, there are still some limitations to this study. Similar to many previous studies, this study examined people with a history of violence before the onset of schizophrenia and those who engaged in violent behaviors after the onset as a whole. However, some studies indicated that the incidence of violent behaviors and treatment effect appeared to be different across different subgroups of schizophrenia. Thus, the group of violent patients with schizophrenia might need to be subdivided in future works. In addition, this study adopted a cross-sectional design to investigate the factors related to violence of patients with schizophrenia, but the causal relationship between these factors and violence could not be determined. Furthermore, some data were obtained from retrospective assessment, which might have resulted in reduced reliability of data, e.g., patients might have under- or over- reported their violent behaviors due to recall bias. Patients with schizophrenia were likely to have impaired metacognitive function (i.e., the ability to understand their own or others people’s psychological/mental status), which might have affected their cognition and report of their past experience.

## Conclusion

This study focused on male patients from the perspectives of socio-demography and clinical characteristics, and used risk assessment tools such as HCR-20 and PCL-R. The study found that the violent and non-violent groups differed in socio-demographics, history of treatment, and psychopathy characteristics. The regression analysis found that young age at violent incident, impulsivity, and relationship instability in HCR-20 as well as the antisocial factor in PCL-R may be predictors of violence. Hopefully, these findings could help us identify patients at a higher risk of violence and provide guidance for the treatment and management of these patients. The present study also indicated the necessity of using both HCR-20 and PCL-R for the assessment of patients with schizophrenia.

## Data availability statement

The raw data supporting the conclusions of this article will be made available by the authors, without undue reservation.

## Ethics statement

The studies involving human participants were reviewed and approved by the Ethics Committees of the Second Xiangya Hospital. The patients/participants provided their written informed consent to participate in this study.

## Author contributions

WG, YG, and QS conceived and designed this study and collected and analyzed the data. WG and YG wrote the manuscript. QS supervised the study and critically revised the manuscript for important intellectual content. JZ and XW contributed to the design and analysis of this study, and assisted in revising the manuscript. All authors contributed to the article and approved the submitted version.
