# Supplementary material for: Characteristics and associated factors of violence in male patients with schizophrenia in China
Source: Front Psychiatry. 2023 Mar 10;14:1106950. doi: 10.3389/fpsyt.2023.1106950 (PMC10036402; doi:10.3389/fpsyt.2023.1106950)
Supplement: Supplementary file 1 [file Table_1.DOCX]

***Supplementary Material***

**Characteristics and risk factors of violence in male patients with schizophrenia in China**

Weilong Guo^†^, Yu Gu^†^, Jiansong Zhou, Xiaoping Wang, Qiaoling Sun^*^

†These authors contributed equally to this work and share first authorship.

*** Correspondence:** Qiaoling Sun**,** [sunqiaoling1@csu.edu.cn](mailto:sunqiaoling1@csu.edu.cn)

1 Supplementary Table

Table S1: Factors included in the logistic regression analysis and their characteristics

| Variate | Type of variate | Option |
| --- | --- | --- |
| Level of education | Binomial | Secondary school and below/high school and above |
| Duration of illness | Binomial | 5 years and above/below 5 years |
| Previous treatment status | Categorical | Untreated/outpatient/inpatient |
| History of alcohol consumption | Binomial | Yes/no |
| History of suicide | Binomial | Yes/no |
| Conceptual disorganization | Ordinal | 1-7 points |
| Mannerism and posturing | Ordinal | 1-7 points |
| Hostility | Ordinal | 1-7 points |
| Suspiciousness | Ordinal | 1-7 points |
| Motor retardation | Ordinal | 1-7 points |
| Young age at violent incident | Ordinal | 0-2 points |
| Relationship instability | Ordinal | 0-2 points |
| Employment problems | Ordinal | 0-2 points |
| Early maladjustment | Ordinal | 0-2 points |
| Prior release or detention failure | Ordinal | 0-2 points |
| Negative attitudes | Ordinal | 0-2 points |
| Impulsivity | Ordinal | 0-2 points |
| Unresponsive to treatment | Ordinal | 0-2 points |
| Plans lack feasibility | Ordinal | 0-2 points |
| Exposure to destabilizers | Ordinal | 0-2 points |
| Lack of personal support | Ordinal | 0-2 points |
| Noncompliance with remediation attempts | Ordinal | 0-2 points |
| Stress | Ordinal | 0-2 points |
| Interpersonal factor | Continuous | (Point) |
| Affective factor | Continuous | (Point) |
| Lifestyle factor | Continuous | (Point) |
| Antisocial factor | Continuous | (Point) |
